# A monoclonal antibody-based sandwich ELISA for measuring canine Thymidine kinase 1 protein and its role as biomarker in canine lymphoma

**DOI:** 10.3389/fvets.2023.1243853

**Published:** 2023-09-22

**Authors:** Hanan Sharif, Sara Saellström, Bhavya Kolli, Kiran Kumar Jagarlamudi, Liya Wang, Henrik Rönnberg, Staffan Eriksson

**Affiliations:** ^1^Alertix Veterinary Diagnostics AB, Stockholm, Sweden; ^2^Department of Anatomy, Physiology, and Biochemistry, Swedish University of Agricultural Sciences, Biomedical Center, Uppsala, Sweden; ^3^University Animal Hospital, Swedish University of Agricultural Sciences, Uppsala, Sweden; ^4^Center of Clinical Comparative Oncology (C3O), Department of Clinical Sciences, Swedish University of Agricultural Sciences, Uppsala, Sweden

**Keywords:** canine TK1 ELISA, monoclonal antibody, immunoassay, serum TK1 activity, serum TK1 concentration, canine lymphoma, blood biomarker, tumor marker

## Abstract

**Introduction:**

Dogs play an important role in society, which increased during the covid epidemics. This has led to a much higher workload for the veterinarians. Therefore, there is a need for efficient diagnostic tools to identify risk of malignant diseases. Here the development of a new test that can solve some of these problems is presented. It is based on serum Thymidine Kinase 1 (TK1), which is a biomarker for cell proliferation and cell lysis.

**Methods:**

Anti-TK1 monoclonal antibodies were produced against two different epitopes, the active site of the TK1 protein and the C-terminal region of canine TK1. The antibodies were developed with hybridoma technology and validated using dot blot, Quartz Crystal Microbalance (QCM) technology, western blots, immunoprecipitation (IP), and enzyme-linked immunosorbent assay (ELISA). Clinical evaluation of Canine TK1 ELISA was done by using sera from 131 healthy dogs and 93 dogs with lymphoma. The two selected Anti-TK1 monoclonal antibodies have Kd values in the range of 10^−9^ M and further analysis with dot and western blots confirmed the high affinity binding of these antibodies. A sandwich Canine TK1 ELISA was developed using the anti-TK1 antibodies, and TK1 concentrations in serum samples were determined using dog recombinant TK1 as a standard.

**Results:**

Serum TK1 protein levels were significantly higher in dogs with lymphoma compared to those in healthy dogs (*p* < 0.0001). Receiver operating curve analysis showed that the canine TK1-ELISA obtain a sensitivity of 0.80, at a specificity of 0.95. Moreover, the Canine TK1 ELISA has a positive predictive value (PPV) of 97%, and the negative predictive value (NPV) of 83%, reflecting the proportion of test results that are truly positive and negative. Furthermore, Canine TK1 ELISA had significantly higher capacity to differentiate dogs with T-cell lymphoma from those with B-cell lymphoma compared to earlier used TK1 activity assays.

**Discussion:**

These results demonstrate that the Canine TK1 ELISA can serve as an efficient tool in the diagnosis and management of dogs with lymphomas.

## 1. Introduction

The typical canine malignant lymphoma patient presents with generalized lymphadenopathy with or without other clinical signs, but far from always this is the situation in the clinic. Malignant lymphoma can be found in many anatomical locations and with varying nonspecific symptoms. Therefore, dogs are usually tested with several additional clinical diagnostic tools, including blood sampling, imaging, cytology, and several biopsies Sometimes exploratory surgery and/or endoscopy to obtain a final diagnosis is necessary. Furthermore, some of the dogs with lymphoma are generally depressed and thus not amenable for many advanced procedures. Thus, a serum biomarker can significantly help in the diagnosis as a routine procedure for canine lymphoma, and both add value in terms of increased animal welfare and avoid several often-costly diagnostic procedures. Moreover, a serum biomarker is well suited as a monitoring tool during medical therapy, where a decrease in the biomarker serum concentration reflects a positive response, and stable or increased level may lead to a change in the protocols or initiation of rescue therapy. The aim of this study was to develop a canine specific Thymidine kinase 1-ELISA, to contribute to the diagnosis of malignant lymphomas in dogs.

Thymidine kinase 1 (TK1) is a pyrimidine salvage pathway enzyme involved in DNA precursors synthesis. TK1 catalyzes the conversion of thymidine-to-thymidine monophosphate (dTMP), that undergoes further phosphorylation’s and finally incorporated into DNA (1). TK1 is upregulated in the late G1 and S phases of the cell cycle and dramatically decreases at the M-phase as a result of a specific degradation pathway (2). However, TK1 activity remains elevated in the G2 and M phases in highly proliferating cells, such as in tumor cells (3). This leads to the release of TK1 protein into the blood stream originating from disrupted tumor cells (4).

TK1 activity-based assays have been used as biomarker for diagnosis and therapy monitoring of different malignancies from many decades in human medicine (5–7). Studies in dogs with hematological malignancies have demonstrated that increased serum TK1 (STK1) activity can be used to detect and monitor these types of diseases in veterinary medicine (8–12). Although the STK1 activity assays are efficient tools for prediction and therapy monitoring of canine hematologic malignancies, difficulties involved in using radio-labeled substrates and need of special equipment have so far led to a limited the use of STK1 activity-based assays.

However, design and development of peptide-based antibodies against human TK1 provided a different approach for TK1 determinations which can overcome the limitations of the activity assays (12–14). Recently, a polyclonal prototype canine TK1 ELISA was developed that could extend the clinical application of TK1 in canine oncology (14, 15). The assay could discriminate between healthy subjects and dogs with different malignancies. Critical issues associated with polyclonal antibodies such as batch-to-batch variation and long-term stability limited further extended use of this biomarker. Therefore, a monoclonal antibody-based Canine TK1 ELISA may significantly improve the clinical applications of TK1.

Here we describe the selection and characterization of monoclonal antibodies (Mabs) that show high affinity for the dog TK1 protein. Furthermore, a Canine sandwich TK1 ELISA was developed using two selected and molecularly defined anti-TK1 antibodies for the determination of canine serum TK1 protein levels. The performance of Canine TK1 ELISA was assessed by using 93 serum samples from dogs with lymphoma. In addition, the assay performance was compared with the established [^3^H] - dThd phosphorylation assay.

## 2. Materials and methods

### 2.1. Canine anti-TK1 antibodies

**Figure 1:**
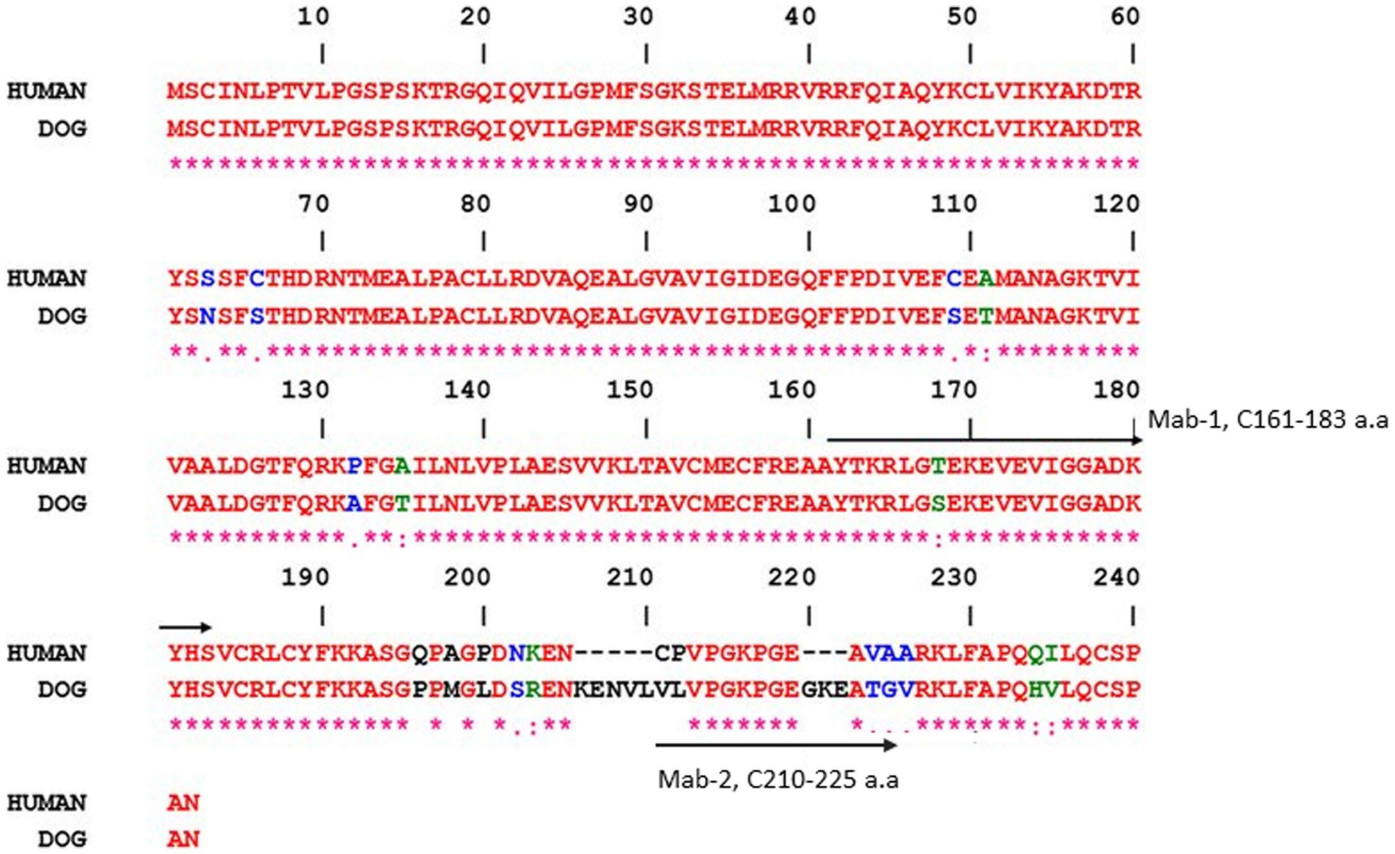
Residues that differ between the two sequences are shown in different colors. The peptide sequences that were used to produce the monoclonal antibodies are indicated by arrows, i.e., mouse monoclonal antibodies Mab-1 (161–183 a.a) and Mab-2 (210–225 a.a). Gene Bank accession numbers are XM_540461 (dog) and KO_2582 (human). The alignments were done using the ESPript program.

The two anti-TK1 antibodies were raised against peptides from different regions of the TK1 sequence; The first antibody (Mab-1) was produced against the active site of the human enzyme, which is a conserved part of the protein in both the human and dog TK1 protein. The other antibody (Mab-2) is directed against the C-terminal of the canine TK1 sequence which has several differences in amino acid sequence as compared to human TK1 as shown in Figure 1. The mouse monoclonal antibodies were produced by GenScript (Piscataway, NJ, United States) using the selected amino acid sequence as previously described (14, 15).

### 2.2. Dog recombinant TK1

Different concentrations of recombinant dog TK1 were used as a standard. The recombinant dog TK1 cDNA was cloned and expressed in *E.coli* and the protein was purified by Ni-Sepharose affinity chromatography as described previously (16).

### 2.3. Electrochemiluminescent dot blot and western blot analysis

#### 2.3.1. Dot blot assay

The ECL dot blot assay was carried out as described previously (12). In brief, 3 μL of recombinant TK1 from different species at varying concentrations, ranging from 20 to 1.25 ng were applied to nitrocellulose membranes (Thermo Fisher Scientific, Sweden). Membranes were blocked with non-fat dry milk 10% (AH diagnostics AB, Sweden) for 1 h and then incubated initially with the hybridoma supernatants, and subsequently with purified monoclonal antibodies overnight at 4°C. Membranes were washed and incubated with the biotinylated second antibody conjugated with horseradish peroxidase directed against mouse IgG (GE healthcare, United Kingdom) for 1 h at room temperature, followed by addition of the ECL reagent. Finally, the signal was detected and quantified by the BIORAD ChemiDoc Imaging System.

#### 2.3.2. Western blot assay

Recombinant dog and human TK1 (10, 5 and 0.5 ng) as well as cytosolic extract from human CEM cells positive and negative for TK1 (12), containing 25 μg of protein, were diluted in the denaturing sample buffer, boiled, and loaded onto a 12% polyacrylamide gel. The gel was run in the SDS running buffer and proteins transferred to PVDF membranes (Millipore, UnitedSA) using a semi-dry slot device. The immunoblotting was carried out as described previously (12).

#### 2.3.3. Immunoprecipitation with Dynabeads

Recombinant dog TK1 (5 ng and 2.5 ng) and diluted 10x serum samples from dogs with different malignancies were separately incubated with the supernatants from hybridoma cultures diluted 10x in the initial screening procedure and subsequently with the purified monoclonal antibodies (4 μg/mL) for 1 h at 4°C and 15 min at 23° C. Dynabeads M-280 (Dynal^®^ sheep anti-mouse IgG, Invitrogen) was prepared according to the manufacturer’s description and incubated with the antigen–antibody complexes for 1 h at 4°C. The beads and the complexes were transferred to a magnetic rack and the supernatants were then analyzed for TK1 activity. The results are presented as % of TK1 activity that bound to the magnetic beads coated with Mab-1 or Mab-2.

### 2.4. Determination of Ka and Kd values

The binding properties of monoclonal TK1 antibodies to recombinant dog TK1 were tested on a QCM biosensor Attana A 200 (Attana AB, Stockholm, Sweden). Recombinant dog TK1 (10, 5 and 2.5 μg/mL) was immobilized onto a LNB carboxyl chip by amine coupling using EDC and S-NHS (17). Assays were performed with a flow rate of 10 μL/mL during amine coupling and 25 μL/mL during binding measurements at 22°C, using HBS-T (10 mM HEPES, 150 mM NaCl, 0.005% Tween 20, pH = 7.4) as a running buffer. Two-fold dilutions of recombinant dog TK1 in HBS-T, were injected over the surface at 10 μL/min and the association and dissociation was monitored for 84 s and 120 s, respectively. HBS-T injection was used as a blank. Sample injections were performed by the C-Fast auto sampler (Attana AB). The binding surface was regenerated after injection by applying 100 mM HCl for 60s and 20 mM NaOH for 30s. Data was processed in the Attana attached software and curve fitting was performed using the TraceDrawer software (Ridgeview instruments). The kinetic parameters including association rate (Ka), dissociation rate (Kd) and the maximum binding capacity (Bmax) were calculated.

### 2.5. Serum sample and specimen handling

The study includes 93 serum samples from dogs diagnosed with lymphoma that were collected from two sources, i.e., 51 samples were purchased from the Flint Animal Cancer Center (Colorado State University) and 42 samples of lymphoma and 131 samples from healthy dogs were collected at the University Animal Hospital, at the Swedish University of Agricultural Sciences (SLU), Uppsala, Sweden, and stored at −20°C until analysis. Information about the clinical staging, grading, and typing of the lymphomas was available for subgroup identification. Data on age, breed, immunophenotype is provided in Supplementary Table 1. The data covering blood count, biochemistry panel, and urinalysis were gathered for all dogs. The dogs with tumors were naive and have not received any prior treatment for cancer.

The group of control dogs were considered healthy based on their medical history, physical examination, hematology, and a basic biochemistry analysis. Most of these subjects were recruited from voluntary blood donor dogs at the University Animal Hospital, SLU, Uppsala, Sweden. Serum samples from dogs with naive lymphoma and from healthy dogs were collected over a 4-year period (2018–2022). At least 1 mL of blood was drawn from each dog and centrifuged within 1 h of collection. The serum samples were stored at −20°C until analysis.

#### 2.5.1. Ethics statement

This project was approved by the Swedish Animal Ethics Committee (ref. no. C12/15) and samples were used only with the owners’ signed consent.

### 2.6. Canine TK1 ELISA

The canine TK1 ELISA was developed by using two selected monoclonal antibodies in a sandwich format. The ELISA procedure is similar to those described previously (11, 12) with slight modifications. Briefly, Mab-1 was immobilized on microtiter plates by Mercodia AB (Uppsala, Sweden) as described (14). Dog sera (60 μL) and different concentrations of recombinant TK1 ranging from 0.120 ng/mL to 2.0 ng/mL were diluted 1:1 with sample dilution buffer (SDB, Alertix AB, Sweden) with recombinant TKI dilutions serving to generate a standard curve. Both serum samples and calibrators were pre-incubated for 1 h at RT and 100 μL of these samples were added to the Mab-1 coated plates and incubated for 2 h. The plates were washed and incubated with biotin labeled Mab-2 (3 μg/mL) for 1 h at RT. The plates were then washed as described above and incubated with 100 μL streptavidin-HRP solution for 30 min (Thermo fisher Scientific, Sweden). After a final wash, the wells were incubated with 100 μL of 1-Step Ultra TMB (Thermo fisher Scientific, Sweden) and the reactions were stopped by adding 100 μL of 1 N, HCL. The absorbance was measured at 450 nm (Tecan M-200+, Switzerland) and samples were analyzed in duplicates. The TK1 protein levels in sera from lymphoma dogs and healthy dogs were determined by using the absorbance of calibrators as a standard in the 4-parameter logistic model provided by SoftMax Pro 7.1.

TK1 protein levels in serum samples were expressed as ng/mL. The canine TK1 ELISA has a limit of blank value (LOB) of 0.02 ng/mL, a limit of detection (LOD) of 0.10 ng/mL and the limit of quantification (LOQ) was 0.15 ng/mL. The cut-off value was set as 2xSD above the mean of TK1 protein levels in healthy dogs. Intra assay CVs with all non-zero calibration points were ≤ 10% and between-run imprecision was ≤15% at concentrations down to 0.12 ng/mL.

### 2.7. The [^3^H]-dThd phosphorylation assay

Serum TK1 activity in all clinical samples were measured by a radiochemical assay using the DEAE filter technique as described previously (PerkinElmer, Waltham-United States) (10). In brief, the reaction mixture contained Tris–HCl pH 7.6, 10 mM; Dithiotreitol, 2 mM; MgCl_2_, 5 mM; NaF, 5 mM; ATP, 5 mM; 5 μM [^3^H]-dThd and 10 μL of serum sample were incubated at 37°C for 1 h. Three aliquots of the reaction mixture were applied to DEAE filter papers which were dried at ambient temperatures. Filters were then washed twice with 1 mM Ammonium Formate for 5 min and the reaction products were eluted for 45 min in 0.1 M HCl and 0.2 M KCl. Finally, the radioactivity was measured by β-scintillation liquid counting and the TK1 activity was expressed as pmol/min/mL. The cut-off value was determined as 2xSD above the mean based on TK1 activity levels in healthy dogs. The TK1 activity assay had an LOD of 0.34 pmol/min/mL and LOQ of 0.9 pmol/min/mL.

### 2.8. Statistical analysis

The distributions of TK1 protein and activity levels in the healthy and lymphoma groups were evaluated for normality using the D’Agostino and Pearson omnibus normality test. The Mann–Whitney U test was used to evaluate the difference between the groups. The Spearman correlation coefficient (*r_s_*) was used to determine the correlation between TK1 protein and other TK1 determination assays. The Receiver operating characteristic (ROC) curves were constructed to evaluate the performance of the TK1 assays. All statistical analysis were performed using Graph Pad Prism 5.0.4 (Graph Pad Software, La Jolla, CA, United States) and MedCalc 17.6 (Seoul, Republic of Korea). The level of statistical significance was set *p* ≤ 0.05.

## 3. Results

### 3.1. Selection and characterization of anti-dog TK1 monoclonal antibodies

**Figure 2:**
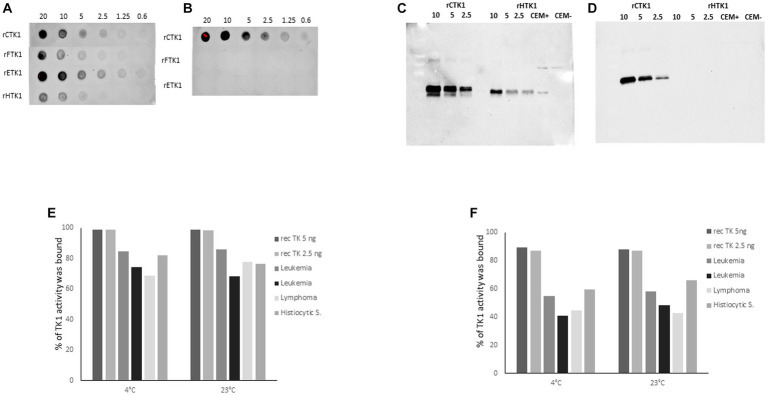
Dot blot results using recombinant enzymes from canine (rCTK1), feline (rFTK1), equine (rETK1) and human (rHTK1) at concentration ranging from 0.6–20 ng. The membranes were developed with **(A)** the monoclonal antibody produced against the active site: Mab-1 (3 μg/mL) and **(B)** the monoclonal antibody produced against the C terminal region: Mab-2 (3 μg/mL). **(C)** Western blot experiments result with 2 μg/mL of Mab-1. **(D)** Western blot results with 2 μg/mL of Mab-2. Immunoprecipitation results with recombinant canine TK1 5 and 2.5 ng and sera from dogs with different malignancies diluted 10x and incubated at 4°C and room temperature in **(E)** with 4 μg/mL of Mab-1, in **(F)** with 4 μg/mL of Mab- 2. The results are presented as the percentage of TK1 protein bound to the magnetic beads coated with anti-mouse IgG.

The reactivity of the hybridoma supernatants and the purified monoclonal antibodies with recombinant TK1 proteins from canine, feline, equine, and human sources at varying concentrations were determined using a dot blot assay (12). Mab-1 raised against the active site of the TK1 showed binding with recombinant TK1 from all species. (Figure 2A). In contrast, Mab-2 produced against the canine C-terminal peptide sequence was only bound to dog recombinant TK1 (Figure 2B).

SDS-PAGE analysis with recombinant TK1 (canine and human) as well as human cell extracts showed a strong binding single band with Mab-1 (Figure 2C). Membrane stained with Mab-2 (2 μg/mL) showed bands with recombinant canine TK1 at varying concentrations, but no bands were observed with human TK1 in agreement with the results using the dot blot assay (Figure 2D). These results demonstrate that the two monoclonal antibodies showed strong reactivity with recombinant dog TK1.

In addition, immunoprecipitation (IP) was performed to determine the reactivity of the antibodies with serum TK1. The IP results with Mab-1 showed that approximately 95% of the recombinant canine TK1 was able to bind to the beads at varying concentrations and experimental conditions (Figure 2E). Moreover, the Mab-1 antibody showed high reactivity with the serum TK1, and 70–85% of serum TK1 was bound to the beads (Figure 2E). The IP results with Mab-2 showed that 90% of the recombinant canine TK1 was able to bind to the beads at 4°C and 23°C (Figure 2F), while 40–60% of TK1 were bound to the beads when serum samples from dogs with leukemia, lymphoma and histiocytic sarcoma were analyzed (Figure 2F). These results demonstrated that the selected monoclonal antibodies have reactivity with both recombinant and serum TK1, but to a different extent, with Mab-1 being somewhat more efficient than Mab-2. However, the detector Mab-2 provided the high specificity for dog TK1 in the sandwich ELISA.

### 3.2. Kinetic characterization of the monoclonal antibodies using the Attana QCM binding technique

The binding constants for the interactions between Mab-1 and Mab-2 with recombinant Canine TK1 were determined as described in the Material and Method section. The analysis showed that the association rate (Ka) of Mab-2 was about two times higher than the Mab 1 (5.1 × 10^5^ and 2.4 × 10^5^, respectively). However, the dissociation rate (Kd) was faster for Mab-1 than that of Mab-2. Consequently, clear interactions in the nanomolar range were observed for canine recombinant TK1 with both Mab-1 (0.5 nM) and Mab-2 (0.3 nM) (Table 1). Thus, Mab-1 and Mab-2 had sufficient binding affinities to detect canine TK1 protein in the nM range, which is clinically relevant for ELISA applications. A similar analysis with serum TK1 was not possible because of the structural complexity of serum TK1 (13).

**Table 1:** Results from the kinetic analysis of Mab-1 and Mab-2 using the Attana QCM binding technique.

Monoclonal antibody	*K_a_* (M^-1S-1^)	*K_d_* (S^−1^)	*K_D_* (nM)	*B*_max_
Mab-1	2.4–3.0^⁎^10 ^5^	6.1^⁎^10^−5^–2.3^⁎^10^−4^	0.3–0.75	34–50
Mab-2	5.0–5.4^⁎^10 ^5^	1.1–1.9^⁎^10^−4^	0.2–0.4	12–16

The kinetic analysis was performed individually for three replicates for canine TK1 and in this table the range between the values are presented. K_a_, association rate constant; K_d_, dissociation rate constant; K_D_, dissociation equilibrium constant; B_max_, binding capacity.

### 3.3. Age distribution in dogs

The age distribution of dogs in the lymphoma group (n = 93) was 2–15 years, with a median age of 8 years, comprising 27 males, 14 females, 31 neutered males, and 21 spayed females. Whereas the age distribution in healthy dogs (n = 131) were between 1–9 years with a median of 3 years, comprising 62 males, 32 females, 19 neutered males, and 2 spayed females. Thus, the dogs in the lymphoma group were about 5 years older than the healthy subjects (*p* ≤ 0.0001).

### 3.4. TK1 protein and TK1 activity levels in healthy dogs and dogs with lymphoma

*Healthy dogs*: The serum TK1 protein levels in healthy dogs were in the range of 0.10 to 0.56 ng/mL. However, 7/131 healthy were identified as outliers using the Grubbs test and these sera were excluded from the statistical analysis. After excluding the 7 dogs, the median TK1 protein of the 124 healthy dogs was 0.13 ng/mL, and the cut off value was 0.22 ng/mL.

The STK1 activity levels in healthy dogs (n = 124) were in the range of 0.4 to 3.2 pmol/min/mL (median = 1.3 pmol/min/mL) and the cut off was 2.3 pmol/min/mL.

To investigate the effect of age on the level of STK1, the healthy group was divided into two subgroups (below and above 4 years). No significant difference was observed regarding both the TK1 activity and TK1 protein levels (Supplementary Figures 1A,B). However, the dogs younger than 4 years showed slightly higher level of TK1 protein compared to dogs above 4 years, Supplementary Figure 1B. The median age of neutered male dogs was significantly higher compared to the intact males and females (Supplementary Figure 1C). Furthermore, the neutered male group showed a significantly lower TK1 protein concentration in comparison to the intact male. Similarly, the female dogs appeared to have a higher median TK1 protein level compared to the neutered dogs, even though this did not reach statistically significance (Supplementary Figure 1D).

**Figure 3:**
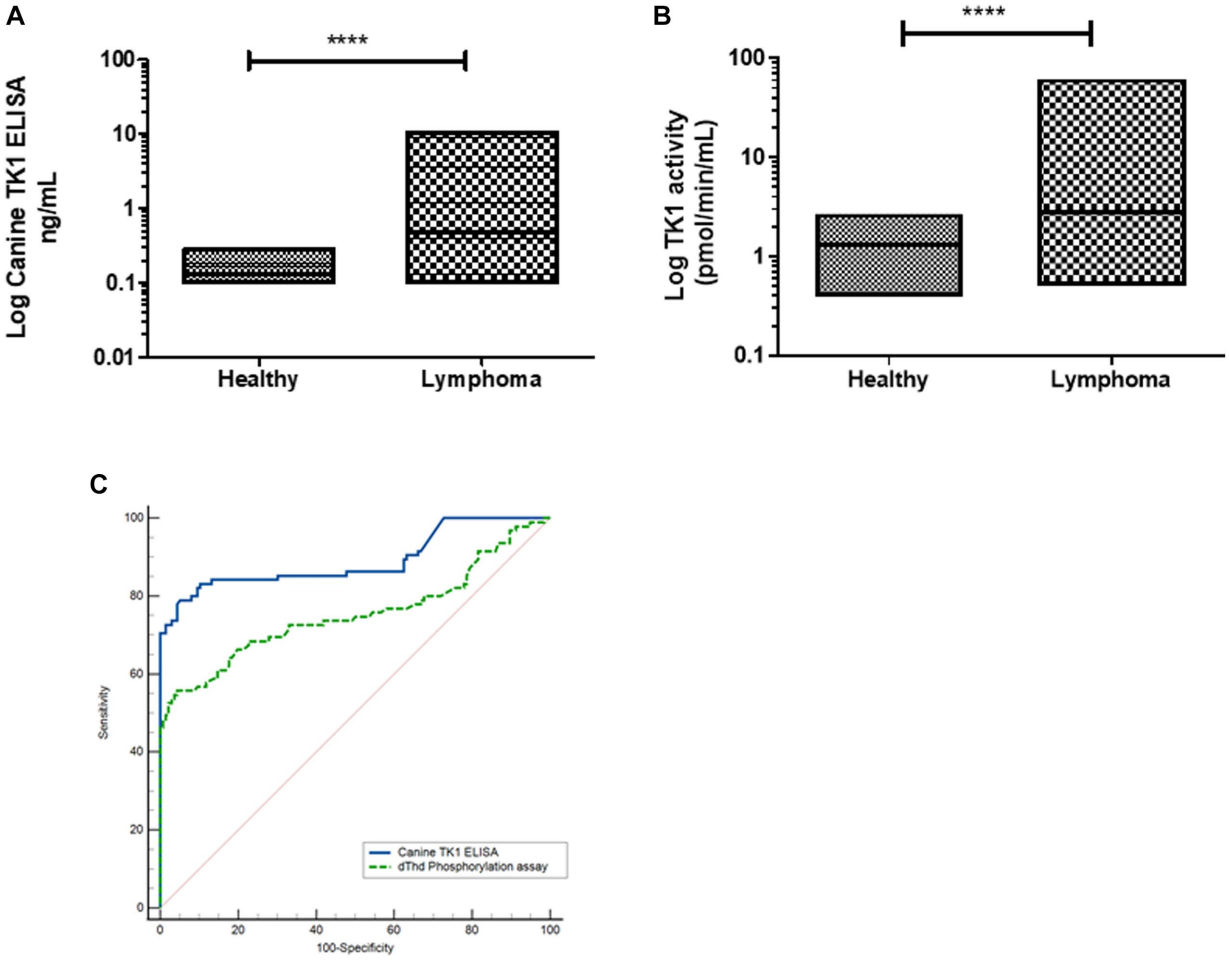
**(A)** Log STK1 protein concentrations measured in sera from healthy dogs (*n* = 124) and dogs with lymphoma (*n* = 93), the lines represent the median values **(B)**. The distribution of Log STK1 activity values in sera from healthy dogs (*n* = 124) and in sera from dogs with lymphoma (*n* = 93), error bars represent the median values. **(C)** Receiver operating characteristic (ROC) curves of the STK1 protein concentrations (blue line) and TK1 activity values (green line) in sera from dogs with tumors and healthy dogs.

*Dogs with lymphoma*: The TK1 concentrations in the lymphoma group were in the range from 0.10 to 10.6 ng/mL (median 0.47 ng/mL), thus significantly higher compared to healthy dogs (Figure 3A). The TK1 activity levels in the healthy group were in the range of 0.50 to 62 pmol/min/mL (median was 2.8 pmol/min/mL, Figure 3B), and there was a significant difference between the healthy and lymphoma groups. ROC curve analysis showed that the Canine TK1 ELISA had an area under the curve (AUC) of 0.88, *p* < 0.0001 (95% confidence interval (CI) 0.82–0.93), with a sensitivity of 0.80 at a specificity of 0.95 (Figure 3C). Further analysis showed an expected positive predictive value (PPV) for Canine TK1 ELISA of 94% and a negative predictive value (NPV) of 80%. In comparison, the dThd phosphorylation assay had an AUC of 0.77 (p < 0.0001, 95% CI 0.70–0.84) with a sensitivity of 0.56 at a specificity of 0.95. The PPV for the TK1 activity assay was 96% and NPV was 74%. In addition, there was a significant difference in the AUC between Canine TK1 ELISA and dThd phosphorylation assay as shown in Figure 3C (*p* = 0.0002).

**Figure 4:**
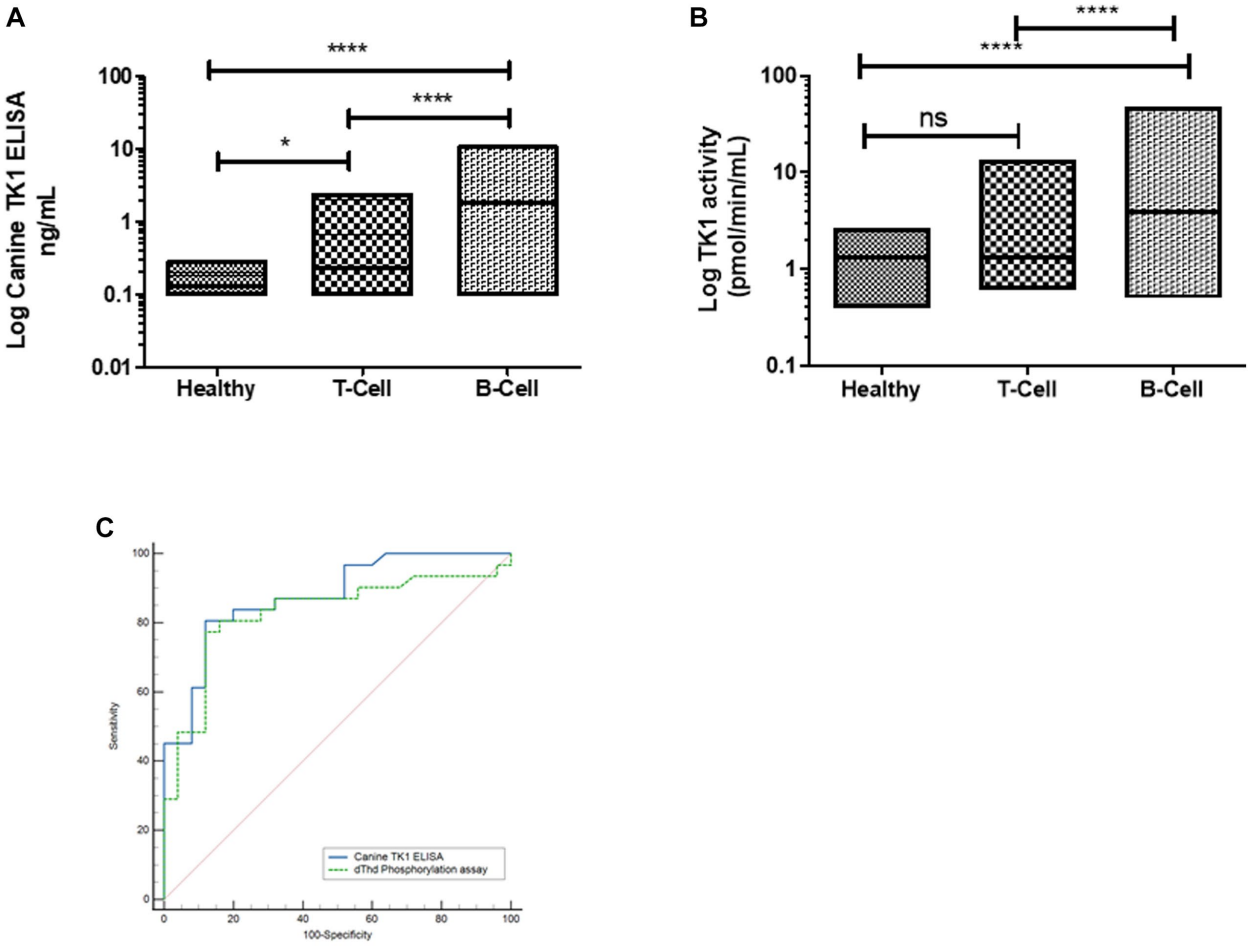
**(A)** Log STK1 protein concentration in sera from healthy dogs (*n* = 124), dogs with T-cell lymphoma (*n* = 25) and B-cell lymphoma (*n* = 32). **(B)** Log STK1 activity levels in healthy dogs, dogs with T-cell lymphoma and B-cell lymphoma. **(C)** ROC curves of STK1 protein values (blue line) and TK1 activity values (green line) to differentiate between dogs with T-cell lymphoma and B-cell lymphoma. * *p* < 0.05; ** *p* < 0.01; *** *p* < 0.001.

**Figure 5:**
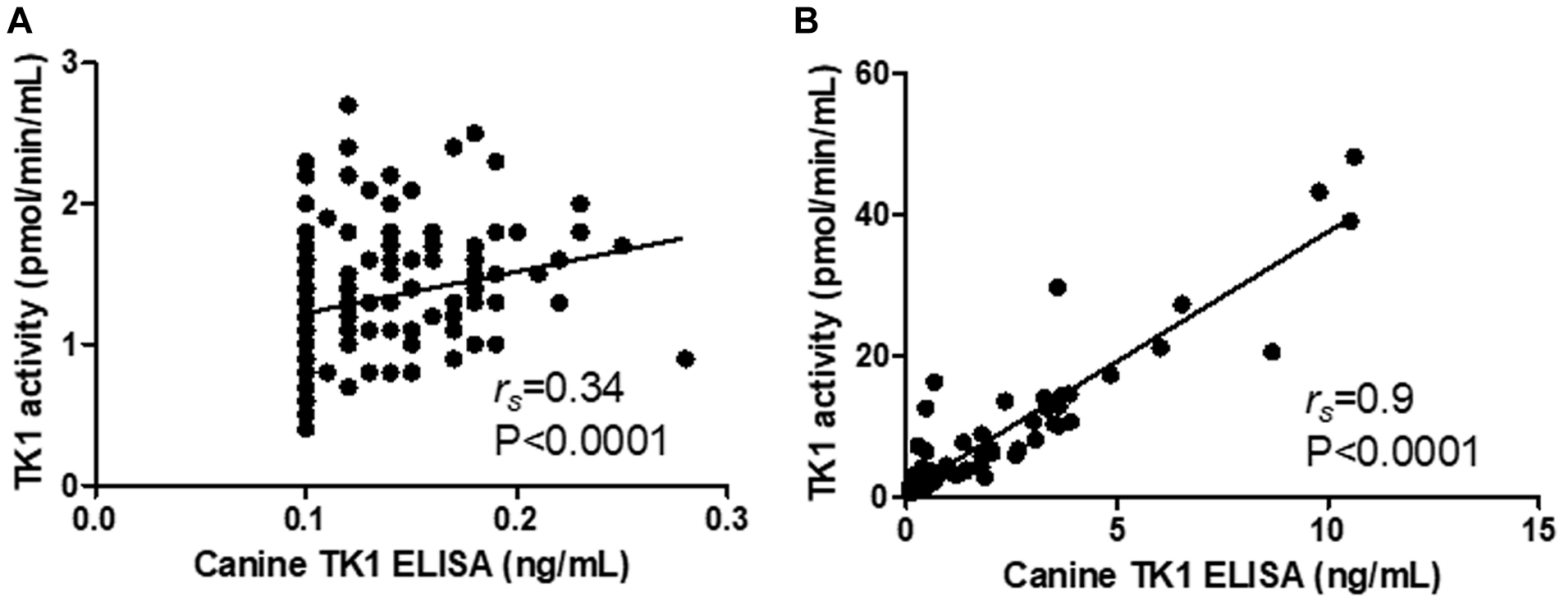
Correlation between the results with the Canine TK ELISA versus the dThd phosphorylation assay in the healthy group **(A)** the lymphoma group **(B)**.

Interestingly, the Canine TK1 ELISA could differentiate T-cell and B-cell lymphoma subgroups from healthy dogs (Figure 4A). The median value of TK1 protein in T-cell lymphoma was 0.23 ng/mL, while the median value in B-cell lymphoma was 1.8 ng/mL. In contrast, the dThd phosphorylation assay could not differentiate T-cell lymphoma from healthy dogs (*p* > 0.05, Figure 4B). The median TK1 activity value in T-cell was 1.3 pmol/min/mL and in B-cell was 3.9 pmol/min/mL. The ROC curve analysis with the canine TK1 ELISA showed a significantly higher AUC compared to the dThd phosphorylation assay in the differentiation of T-cell lymphoma from B-cell lymphomas (*p* = 0.028; Figure 4C). In case of healthy dogs, a weak correlation was found between canine TK1 ELISA and dThd phosphorylation assay (*r_s_* = 0.34, Figure 5A), while the correlation was significantly higher between the assays in the lymphoma group (rs = 0.9) (Figure 5B).

### 3.5. Comparison with the DiviTum TK1^®^ and AroCell TK 210 ELISA^®^ assays

A subset of serum samples from dogs with lymphoma (*n* = 25) and healthy dogs (*n* = 20) were evaluated with four different TK1 assays, two of which were based on TK1 activity measurements (DiviTum assay Biovica, Uppsala, Sweden and dThd phosphorylation assay) and two immunoassays (Canine TK1 ELISA and AroCell TK 210 ELISA, Arocell, Bromma, Sweden). Both the TK1 activity determined with DiviTum assay as well as dThd phosphorylation assay showed significantly higher levels compared to healthy controls and similar results were observed with Canine TK1 ELISA also (Supplementary Figures 2A–C). However, the ROC curve analysis demonstrates that Canine TK1 ELISA had higher sensitivity compared to the two activity assays in differentiating lymphoma from the healthy dog subgroups (Supplementary Figures 2D–F). The AroCell TK 210 ELISA could not detect any TK1 protein in extracts from the lymphoma or the healthy dogs. The Divitum and Arocell assays were used per manufacturers’ instructions.

Furthermore, significant correlations were observed between the Canine TK1 ELISA and the DiviTum assay results (*r_s_* = 0.63, *p* < 0.0001; Supplementary Figure 3A), and between the Canine TK1 ELISA and the dThd phosphorylation assay results (*r_s_* = 0.81, *p* = 0.0001; Supplementary Figure 3B). This was also observed with the dThd phosphorylation assay and the DiviTum assay (*r_s_* = 0.7, *p* < 0.0001; Supplementary Figure 3C) when assaying the lymphoma subgroups (*N* = 25).

## 4. Discussion

The gold standard of canine lymphoma diagnosis is cytology and/or biopsy of suspicious lesions. All diagnostics have a risk of false positive and false negative results and often the clinical appearance of canine lymphoma is not obvious at first presentation. Adding a serum biomarker with high sensitivity and specificity for canine lymphoma would add significant value to the diagnostic standard procedure. Moreover, since blood sampling almost always is part of the examination of dogs with symptoms TK1-ELISA determinations may lead to less need for additional testing.

Immunophenotyping to distinguish between B- or T-cell lymphomas usually include multiple biopsies and immunohistochemistry, flow-cytometry, as well as the PARR PCR test, targeting the CDR3 region of T-cell receptor. All of these tests have different challenges and (can) vary in availability. Thus, having the possibility to discriminate between a B- versus T-cell phenotype with TK1-ELISA adds value in the clinical situation.

This is the first report of the development and application of a sandwich monoclonal TK1 ELISA with sera from healthy dogs and dogs with lymphoma. Earlier studies with a poly/monoclonal antibody-based dog TK1ELISA demonstrated similar sensitivity as the activity assay for prognosing and therapy monitoring of hematological malignancies (14). However, there were problems with the reactivity of polyclonal antibodies such as batch-to batch variation and stability. To avoid this, a monoclonal antibody-based ELISA that could improve the production and clinical applicability of a TK1 assay in routine diagnostics was designed and validated. In the present study we described the development and initial clinical evaluation of monoclonal antibody-based sandwich canine TK1 ELISA. This assay uses antibodies that recognize different epitopes on the TK1 protein which increase its sensitivity as well as specificity. Furthermore, it allows adaptation to clinically used automated platforms. The antibody characterization showed that antibodies raised against the C-terminal of TK1 leads to canine specificity with no cross reactivity with TK1 from other species.

Serum TK1 is a biomarker that reflects accelerated cell proliferation and cell lysis both in normal and tumor cells. The STK1 activity levels have been found to be up regulated in dogs with different malignancies but are often very low or undetectable in healthy dogs (9–11). In the present study, about 20% of healthy dogs showed TK1 protein concentration lower than the detection limit.

Development of a specific routine assay for TK1 ELISA may improve health screening tests for older dogs and lead to early detection of malignant diseases (11). However, a transient increase of STK1 have been detected in several infectious and inflammatory diseases (8, 18). Therefore, when evaluating elevated levels of TK1 as a risk factor for malignancies, markers specific for inflammatory conditions could increase the diagnostic accuracy (15, 19, 20).

This study also showed that the Canine TK1 ELISA was able to aid in the differentiation between the T-cell and B-cell within the lymphoma group, which is of large clinical importance for the type of treatment and prognosis of dogs with malignant lymphoma. Furthermore, the capacity of this assay in monitoring treatments in most lymphoma cases is a major advantage since it may ensure that the side effects and costs are motivated. This as, high grade T-cell lymphomas normally imply a worse prognosis and moreover often suggest using other drugs and protocol complimentary to the standard CHOP mostly used in B-cell lymphoma (21). Finally, the T-cell phenotype are often associated with a larger degree of drug resistance (21). Yet to be proven is if the magnitude of serum TK1 concentration determined by this Canine TK1 ELISA upon initial presentation of the patient can be prognostic. This has been reported in humans for TK1 in both breast cancer and NHL and would add value for the individual dog (owner) as well as the treating veterinarian (22, 23).

Future other possible applications of TK1 could be to address level of tissue trauma or amount of hypoxia in intensive care cases, then reflecting TK1 leakages from normal cells.

The results specifically presented here demonstrate that the monoclonal canine TK1 ELISA may serve as an efficient tool to estimate the increased cell proliferation responsible for the aggressiveness and type of canine lymphoma, which can aid cancer management in veterinary medicine.

## Data availability statement

The original contributions presented in the study are included in the article/supplementary material, further inquiries can be directed to the corresponding author.

## Ethics statement

The animal studies were approved by the Swedish Animal Ethics Committee (ref. no. C12/15) and samples were used only with the owners’ signed consent. The studies were conducted in accordance with the local legislation and institutional requirements. Written informed consent was obtained from the owners for the participation of their animals in this study.

## Author contributions

HR, HS, SS, BK, KJ, LW, and SE substantially contributed to the design of the work as well as interpretation of data for the work, revising it critically for important intellectual content, provided approval for publication of the content and agreed to be accountable for all aspects of the work in ensuring that questions related to the accuracy or integrity of any part of the work are appropriately investigated and resolved. HS drafted the manuscript. SS sampled, provided, clinically phenotyped, and treated the majority of the included samples in the study. All authors contributed to the article and approved the submitted version.

## Funding

This study was supported by funds from the Swedish Research Council (SE), the Swedish University of Agricultural Sciences future platform SLU Future animals, nature, and health (SS and HR) and the Johansson Family Swedish Boxer Club Cancer Donation (SS and HR). Some minor funding from Alertix Veterinary Diagnostic AB to support the completion of the experiments has been obtained. The funders were not involved in the study design, collection, analysis, interpretation of data, the writing of this article or the decision to submit it for publication.

## Conflict of interest

HS, BK, and SE, were employed by Alertix Veterinary Diagnostics AB. KJ was employed by AroCell AB. SE is a co-inventor of a TK1 activity patent licensed to DiaSorin Inc. (and several patents owned by AroCell AB). He is a co-founder, shareholder, and consultant to the company AroCell AB, Uppsala. There is a U.S. patent pending for the antibodies and assay procedures described in this manuscript: Determination of Non-Human Mammal TK1 protein levels, with KJ, HR, and SE as inventors. HR is member of the scientific advisory board and shareholder of Alertix. This does not alter the authors' adherence to all the Frontiers Journal’s policies on sharing data and materials.

The remaining authors declare that the research was conducted in the absence of any commercial or financial relationships that could be construed as a potential conflict of interest.

## Publisher’s note

All claims expressed in this article are solely those of the authors and do not necessarily represent those of their affiliated organizations, or those of the publisher, the editors and the reviewers. Any product that may be evaluated in this article, or claim that may be made by its manufacturer, is not guaranteed or endorsed by the publisher.

## Supplementary material

The Supplementary material for this article can be found online at: https://www.frontiersin.org/articles/10.3389/fvets.2023.1243853/full#supplementary-material
