# Supplementary material for: A monoclonal antibody-based sandwich ELISA for measuring canine Thymidine kinase 1 protein and its role as biomarker in canine lymphoma
Source: Front Vet Sci. 2023 Sep 22;10:1243853. doi: 10.3389/fvets.2023.1243853 (PMC10557065; doi:10.3389/fvets.2023.1243853)
Supplement: Supplementary file 1 [file Table_1.pdf]

Sample ID	Age	Breed	Immunophenotype
T1	14	Golden retriever	T cell lymphoma
T2	5	Golden retriever	T cell lymphoma
T3	5	Boxer	T cell lymphoma
T4	6	Boxer	T cell lymphoma
T5	9	Boxer	T cell lymphoma
T6	8	Shorthaired dachshound	T cell lymphoma
T7	7	Golden retriever	T cell lymphoma
T8	13	Welsh corgi	T cell lymphoma
T9	7	Boxer	T cell lymphoma
T10	7	Labrador retriever	T cell lymphoma
T11	5	Golden retriever	T cell lymphoma
T12	10	Shih tzu	T cell lymphoma
T13	10	Golden retriever	T cell lymphoma
T14	8	Mixed breed	T cell lymphoma
T15	3	Mixed breed	T cell lymphoma
T16	9	Mixed breed	T cell lymphoma
T17	3	Mixed breed	T cell lymphoma
T18	6	German shepherd	T cell lymphoma
T19	11	English setter	T cell lymphoma
T20	11	Boxer	T cell lymphoma
T21	9	Boxer	T cell lymphoma
T22	10	Hungarian Vizla	T cell lymphoma
T23	9	Flat coated retriever	T cell lymphoma
T24	8	German shepherd	T cell lymphoma
T25	6	Boxer	T cell lymphoma
B1	14	Nova scotia duck tolling retriever	B cell lymphoma
B2	6	Podengo portugues	B cell lymphoma
B3	11	Bernese moutaindog	B cell lymphoma
B4	7	Alaskan malamute	B cell lymphoma
B5	2	Mixed breed	B cell lymphoma
B6	5	Golden retriever	B cell lymphoma
B7	9	Mixed breed	B cell lymphoma
B8	6	Siberian husky	B cell lymphoma
B9	13	Mixed breed	B cell lymphoma
B10	12	Labrador retriever	B cell lymphoma
B11	12	Toy fox terrier	B cell lymphoma
B12	5	Mixed breed	B cell lymphoma
B13	7	Golden retriever	B cell lymphoma
B14	8	Mixed breed	B cell lymphoma
B15	7	Vizsla	B cell lymphoma
B16	7	Golden retriever	B cell lymphoma
B17	6	labrador retriver	B cell lymphoma
B18	12	Golden retriever	B cell lymphoma
B19	9	Doberman pinscher	B cell lymphoma
B20	13	Mixed breed	B cell lymphoma
B21	3	Aktia	B cell lymphoma
B22	11	Labrador retriever	B cell lymphoma
B23	13	staffordshire terrier	B cell lymphoma
B24	10	Golden retriever	B cell lymphoma
B25	10	Mixed breed	B cell lymphoma
B26	9	English setter	B cell lymphoma
B27	6	Rottweiler	B cell lymphoma
B28	10	Golden Retriever	B cell lymphoma
B29	7	Golden Retriever	B cell lymphoma
B30	9	Bulldog (French)	B cell lymphoma
B31	13	Tibetan Terrier	B cell lymphoma
B32	9	Afghan Hound	B cell lymphoma
