# Supplementary figures and images for: A monoclonal antibody-based sandwich ELISA for measuring canine Thymidine kinase 1 protein and its role as biomarker in canine lymphoma
Source: Front Vet Sci. 2023 Sep 22;10:1243853. doi: 10.3389/fvets.2023.1243853 (PMC10557065; doi:10.3389/fvets.2023.1243853)

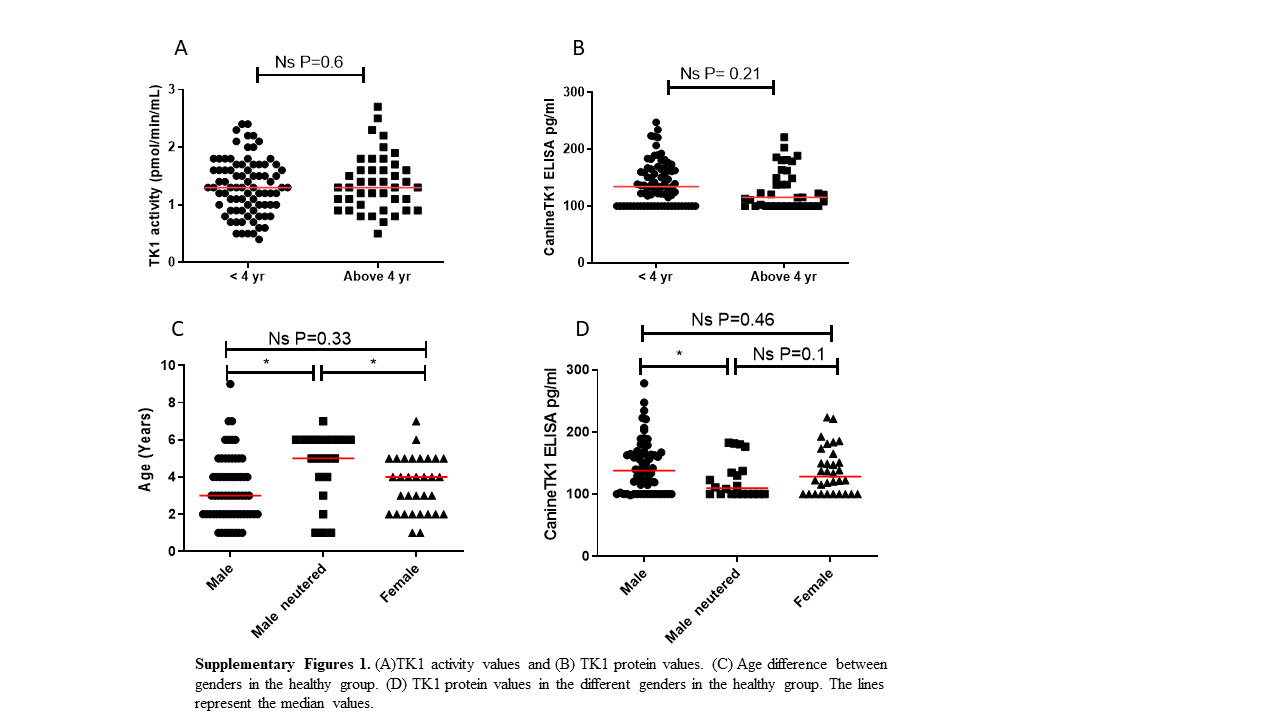

Supplement: Supplementary file 2 [file Image_1.TIF]

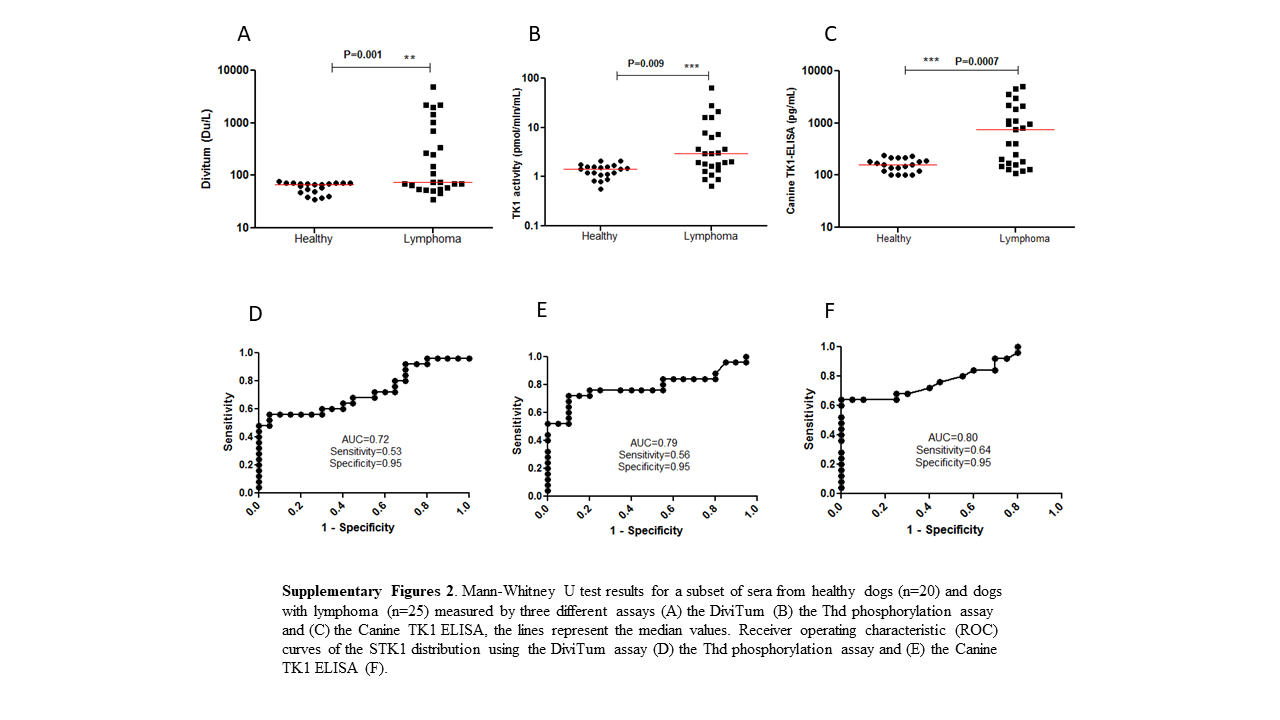

Supplement: Supplementary file 3 [file Image_2.TIF]

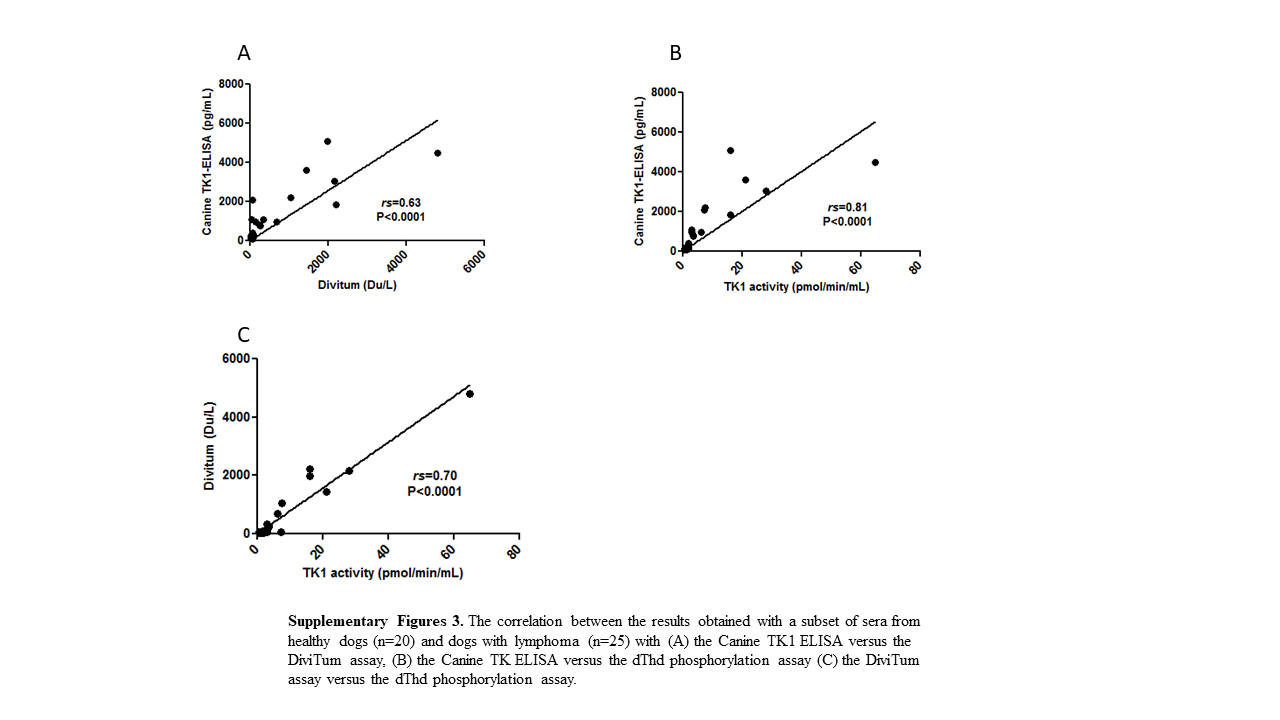

Supplement: Supplementary file 4 [file Image_3.TIF]
